# Enhancing HIV Treatment Access and Outcomes Amongst HIV Infected Children and Adolescents in Resource Limited Settings

**DOI:** 10.1007/s10995-016-2074-1

**Published:** 2016-08-11

**Authors:** Ameena Ebrahim Goga, Yagespari Singh, Michelle Singh, Nobuntu Noveve, Vuyolwethu Magasana, Trisha Ramraj, Fareed Abdullah, Ashraf H. Coovadia, Sanjana Bhardwaj, Gayle G. Sherman

**Affiliations:** 1 Health Systems Research Unit, South African Medical Research Council, Francie van Zyl Drive, Parrowvallei, Cape Town, 7505 South Africa; 2 Department of Paediatrics, University of Pretoria, Private bag X20, Hatfield, Pretoria, 0028 South Africa; 3 South African National AIDS Council, Pretoria, South Africa; 4 Department of Paediatrics and Child Health, Faculty of Health Sciences, University of the Witwatersrand, Johannesburg, South Africa; 5 United Nations Childrens Fund, Pretoria, South Africa; 6 National Institute of Communicable Diseases, National Health Laboratory Services, Modderfontein Road, Sandringham, Johannesburg, South Africa

**Keywords:** Adolescent, Paediatric ART access, Paediatric HIV, ARV uptake, ARV coverage, Paediatric HIV, Continuity of care, Paediatric HIV treatment access, PMTCT effectiveness, SAPMTCTE

## Abstract

*Introduction* Increasing access to HIV-related care and treatment for children aged 0–18 years in resource-limited settings is an urgent global priority. In 2011–2012 the percentage increase in children accessing antiretroviral therapy was approximately half that of adults (11 vs. 21 %). We propose a model for increasing access to, and retention in, paediatric HIV care and treatment in resource-limited settings. *Methods* Following a rapid appraisal of recent literature seven main challenges in paediatric HIV-related care and treatment were identified: (1) lack of regular, integrated, ongoing HIV-related diagnosis; (2) weak facility-based systems for tracking and retention in care; (3) interrupted availability of dried blood spot cards (expiration/stock outs); (4) poor quality control of rapid HIV testing; (5) supply-related gaps at health facility-laboratory interface; (6) poor uptake of HIV testing, possibly relating to a fatalistic belief about HIV infection; (7) community-associated reasons e.g. non-disclosure and weak systems for social support, resulting in poor retention in care. *Results* To increase sustained access to paediatric HIV-related care and treatment, regular updating of Policies, review of inter-sectoral Plans (at facility and community levels) and evaluation of Programme implementation and impact (at national, subnational, facility and community levels) are non-negotiable critical elements. Additionally we recommend the intensified implementation of seven main interventions: (1) update or refresher messaging for health care staff and simple messaging for key staff at early childhood development centres and schools; (2) contact tracing, disclosure and retention monitoring; (3) paying particular attention to infant dried blood spot (DBS) stock control; (4) regular quality assurance of rapid HIV testing procedures; (5) workshops/meetings/dialogues between health facilities and laboratories to resolve transport-related gaps and to facilitate return of results to facilities; (6) community leader and health worker advocacy at creches, schools, religious centres to increase uptake of HIV testing and dispel fatalistic beliefs about HIV; (7) use of mobile communication technology (m-health) and peer/community supporters to maintain contact with patients. *Discussion and Conclusion* We propose that this package of facility, community and family-orientated interventions are needed to change the trajectory of the paediatric HIV epidemic and its associated patterns of morbidity and mortality, thus achieving the double dividend of improving HIV-free survival.

## Significance

Drawing from research, this commentary provides a perspective on ways to increase access to paediatric antiretroviral therapy in resource-limited settings. It presents a possible model to integrate seven facility, community and family-based interventions and implement them as a package. This is critical at a time when global efforts to improve paediatric antiretroviral access are gaining momentum.

## Introduction

In 2009 approximately 5.1 million young people aged 15–24 years were living with HIV globally (UNICEF Report 2014). By 2011 although the number of new paediatric HIV infections had decreased substantially, 300,000 new paediatric infections were detected at birth and 3.4 million children were estimated to be living with HIV. More than 90 % of these children live in resource-constrained settings in Sub-Saharan Africa (UNAIDS and WHO 2011). In 2011–2012, in the 22 countries with the highest burden of antenatal HIV infection only 31 % (29–33 %) of children aged 15 years and below, eligible for antiretroviral (ARV) treatment received it; additionally the percentage increase in children accessing antiretroviral therapy was approximately half that of adults (11 % vs. 21 %) (WHO et al. 2013). Successful prevention or early, regular treatment of paediatric HIV infection, is critical to reduce short-and long-term morbidity and mortality in children aged 0–18 years (Bernays et al. 2014; Triant et al. 2013).

## Methods

Following a rapid appraisal of recent literature mainly from high HIV prevalence settings, we identified seven specific gaps to optimal paediatric HIV care and treatment and classified these into three broad groups, viz.

1. Supply-related gaps at facility level:
  - *Lack of regular, integrated, ongoing HIV-related diagnosis*: A national study in South Africa demonstrated that although more than 70 % of nurses acknowledged the importance of integration between HIV testing and routine immunization/child health services, 69 % of child health nurses thought this was not feasible, due to staff shortages, and time constraints (Woldesenbet et al. 2012). Lack of time seems to pervade health systems: time and financial constraints, and lack of training inhibited the provision of routine HIV testing even in developed country settings such as the USA (Simmons et al. 2011).
  - *Weak facility-based systems to track HIV positive children, track contacts, facilitate disclosure and increase retention in care*: A national study in South Africa revealed that only 46 % of HIV-infected children were referred with letters and appointments; 38 % with only letters and only 57 % of health facilities reported monitoring the uptake of these referrals (Woldesenbet et al. 2012). Furthermore a multi-country meta-analysis demonstrated how delayed diagnosis and treatment initiation affected physiological status: median cluster differentiation four (CD4) cell count was low at antiretroviral therapy initiation amongst all children aged ≥5 years [206 (boys) and 226 (girls) cells/mm^3^] and significantly lower for children in lower and upper middle income countries (median of 183 cells/mm^3^ in girls and 191 cells/mm^3^ in boys) compared with the USA (Koller et al. 2015). Additionally in children <5 years, severe immunodeficiency was documented in 56–64 % of children initiating antiretroviral therapy in resource-limited settings compared with 22 % in the USA (Koller et al. 2015). Apart from late treatment initiation, retention of HIV-exposed children in care is low, with high loss to follow-up (19–89 %) at various points in the diagnostic and treatment cascade (Kalembo and Zgambo 2012). In India 63 % of HIV exposed infants were lost to follow-up after 1 year (Kumar et al. 2015). A Kenyan study illustrated that of the 213 HIV-exposed children eligible for continued care, 68 % presented after age 2 months, 65 % dropped out before the 18 month follow-up and 43 % of these drop-outs occurred within the first two months of age (Hassan et al. 2012). Infant dropout along the care and treatment cascade was associated with younger mothers, poor healthcare worker training and knowledge, poor caregiver understanding of early infant diagnosis (EID), absence of social support, poor physical access to health facilities, stock-outs of infant DBS cards and delayed availability of HIV test results (Hassan et al. 2012). Late antenatal care presentation (≥28 weeks gestation) due to high transport costs, lack of knowledge or poor maternal health is also a strong predictor of loss to follow-up (Chetty et al. 2012).
  - *Interrupted availability of dried blood spot cards* (*expiration/stock outs*): Children under the age of 18 months rely on infant heel prick blood draw onto dried blood spots (DBS) cards and subsequent laboratory diagnosis using polymerase chain reaction (PCR). African countries have expanded laboratory coverage for PCR testing, and in some low to middle income countries, non-governmental organisations such as Riders for Health assist with specimen transportation to the laboratory. South African data demonstrated that interrupted availability of infant test cards for drawing DBS was mainly linked with poor systems for stock control, or delayed supply of pre-packed supplies for DBS blood draw (Woldesenbet et al. 2012).
  - *Poor quality control of rapid HIV testing*: At 18 months and beyond, rapid HIV tests are used for paediatric HIV diagnosis. A study conducted in 455 sites (primary health clinics, community health centres and hospital gateway clinics) in Limpopo Province, South Africa, identified inadequate training, frequent rotation of staff, lack of supervision and on-site quality control, poor adherence to standard operating procedures (SOP) and poor stock control as reasons for incorrect HIV Rapid test results (Adrian Puren, personal communication, 11 March 2015).
2. *Supply-related gaps at health facility-laboratory interface*: A national study in South Africa (2010) revealed that a daily specimen transport system was available in 60–90 % of health facilities (Woldesenbet et al. 2012), and that not all facilities are able to return infant HIV test results within 4 weeks (Woldesenbet et al. 2012). Delayed return of results has been linked with the lack of telephone lines, fax lines, internet and computers at facility level, necessitating the use of paper-based and physical transport systems for return of results (Hassan et al. 2012; Chetty et al. 2012).
3. Demand-related and retention gaps at community level that hinder uptake of care along the paediatric HIV testing and treatment cascade viz. HIV testing, acceptance of results, initiation of treatment and regular follow-up:
  - *Poor uptake of HIV testing, possibly relating to a fatalistic belief about HIV infection*: In a South African study, only 47 % of HIV positive mothers attending primary healthcare facilities at age 6 weeks requested infant HIV testing at the same facility; however acceptance of HIV testing increased to >98 % when research nurses offered infant HIV testing to all caregivers at these sites (Goga et al. 2012). Qualitative research amongst parents/caregivers in Mozambique showed that traditional beliefs in spiritual causes of illness, disbelief of HIV test results and the lack of healthy HIV positive role models created a psychological impasse that prevented self-initiated timely and continued access to care (De Schacht et al. 2014).
  - *Community-associated reasons e.g. non-disclosure of HIV positive status and weak systems for social support resulting in poor retention in care and low adherence to treatment*: Reasons for loss to follow-up are protean but included fear, stigma, home deliveries, low socio-economic status and lack of transport to access health facilities (Kalembo and Zgambo 2012). Non-disclosure of HIV positive status has also been associated with poor adherence (O’Malley et al. 2015).

## Results: Proposed Model to Enhance Treatment Access

**Fig. 1:**
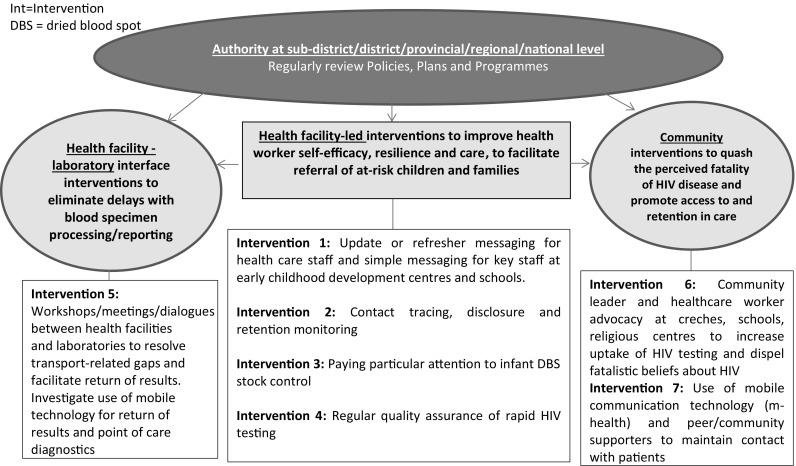
Proposed model including 7 main interventions to enhance HIV diagnosis, treatment access and retention in care amongst HIV infected children and adolescents

Appropriate policies, plans, and programmes are critical for determining infant HIV exposure, testing children for HIV infection, returning HIV test results, referring children and their families into HIV-related care and retaining HIV-infected children and their families in care. Given the rapid pace at which new knowledge in the field of HIV is emerging, regular updating of policies, review of inter-sectoral plans (at facility and community levels) and evaluation of programme implementation and impact (at national, subnational, facility and community levels) is a critical element of care and thus, non-negotiable. Additionally we recommend the intensified implementation of seven main interventions (Fig. 1).

### Health Facility-Led Interventions to Improve Healthcare Worker Self-Efficacy, Resilience and Care to Facilitate Referral of At-Risk Children and Families

#### Intervention 1: Update or Refresher Messaging for Healthcare Staff and Simple Messaging for Key Staff at Early Childhood Development Centres and Schools

- All levels of healthcare staff, including community-based cadres and lay health counsellors, should be reminded to encourage the provision of regular, targeted HIV-related care, integrated into routine adult, child and adolescent healthcare, thus eliminating missed opportunities for preventing, diagnosing or treating paediatric HIV infection. Coverage and timeliness of HIV-related messaging for healthcare staff can be increased by making HIV testing and treatment guidelines freely available on computers and smartphones (Peter et al. 2016). Through a public–private partnership South African HIV treatment guidelines are available through Google Play (Android devices) or the App Store (iOS devices) and preliminary data show that 6006 healthcare workers were empowered through the app and more than 200,000 people accessed the application (www.openmedicineproject.org) during its first month of implementation. The benefits of mobile technology was also demonstrated in Kenya where short reminder cell phone messages to healthcare providers maintained knowledge and enabled the implementation of guidelines more successfully, compared with once off training only (Jones et al. 2012). Increased exposure to information could create new opportunities for HIV-related care to become habit/routine. As women and adolescents generally access reproductive health care, antenatal care, maternal health or contraceptive services, the continued provision of HIV testing during each of these visits is critical. Once HIV testing at these visits becomes habitual, the perceived shortage of time to conduct HIV testing will be diluted. Each healthcare facility should also identify its individual targets for uptake of HIV testing, turn-around-time for return of results and retention in care, and monitor these monthly using tools akin to run charts.

(b) All or selected staff in early childhood development centres, orphanages and schools, should be trained in using algorithms or guidelines that help identify children or families needing referral to healthcare centres for counselling and additional care. Criteria could include orphans, recent maternal or paternal death from HIV infection or TB, family contact with TB, poor weight gain or looks malnourished, sores in the mouth or sores that do not heal.

#### Intervention 2: Contact Tracing, Disclosure and Retention Monitoring

- We recommend that health care workers include a discussion about contact tracing of partners, children, siblings and networks (HIV counselling and testing plus, HCT+) during every interaction with HIV positive adults and children. A summary paper from Canada demonstrates the success of three approaches to contact tracing, namely patient-led, healthcare provider-led or public health authority led contact tracing (Broeckaert and Haworth-Brockman 2014). Healthcare provider-led contact tracing in a Canadian setting was acceptable to clients. Contact tracing of 63 % of notified partners resulted in 14–26 % being diagnosed as HIV positive, identified new infections, increased HIV testing rates amongst untested people and averted high HIV-related costs through early diagnosis. A network and enhanced network approach to identifying contacts has also been used: In the network approach the index case identified everyone in their social networks who may benefit from HIV testing. The number of newly diagnosed HIV positive people quadrupled using this approach. In the enhanced network approach HIV testing services were established in locations frequented by the index case’s networks. This significantly increased the HIV positivity rate for contacts traced in Vancouver. In resource-limited settings an adaptation of network approach could be used: ‘Health promotion’ referral cards could be provided to the index case for all people, including children, in the index case’s network. This ‘health promotion’ package should include HIV testing. Additionally the enhanced network approach could be used to set up community-based HIV testing or HIV testing awareness in the community, especially targetting key populations such as men. HIV testing sites could be set up in places that index cases frequent e.g. outside shebeens, near football matches. For women, community-based HIV testing or HIV testing awareness could be set up near stores selling sanitary supplies. Males and females can also be covered by HIV testing or awareness sites at colleges, technical school or universities. If community based HIV testing and counselling is not practical or ethical then HIV testing awareness could be provided with referral to a health centre using a health promotion referral card.
- Self-testing amongst children older than 12 years has been suggested as an intervention, but Strode et al. (2013) suggest that it only be supported if in the child’s best interest and if support systems are available for pre-and post-test counselling.
- Age-appropriate interventions e.g. cartoon booklets used to train caregivers and healthcare providers facilitate disclosure and increased the self-efficacy of both groups, improving child hopefulness and adherence, and should thus be considered and tested (O’Malley et al. 2015).
- Most countries have facility-based registers and patient-held road to health charts/cards/booklets (RTHB) for monitoring the health of a child under the age of 5 years (Kitenge and Govender 2013). Studies have shown that the RTHB is under-utilized by both healthcare workers (HCWs) and mothers (Cloete et al. 2013). Field experiences in Malawi, Uganda, Cote de ‘Ivoire and the Democratic Republic of the Congo illustrate the benefit of longitudinal registers which could be used to track patient clinic attendance (Doherty et al. 2016). Coupled with community linkage staff e.g. community health workers who trace patients with missed visits, longitudinal facility-held registers and patient-held cards e.g. RTHB could greatly increase early diagnosis, early treatment and retention in care. Some countries have established electronic registers. These electronic tools need to be used on-site for individual-level patient monitoring, and as a tool for distal monitoring of programme uptake and success.

#### Intervention 3: Paying Particular Attention to Infant DBS (Dried Blood Spot) Stock Control

On-site interventions should include systems for stock control and ordering of pre-packed DBS kits containing all the items required to perform a single test, allowing quicker testing and preventing missed opportunities because there is no stock of a particular item (e.g. a needle for heel pricks) (Ghadrshenas et al. 2013).

#### Intervention 4: Regular Quality Assurance of Rapid HIV Testing Procedures

Regular quality assurance of testing procedures and environments is recommended (The Interagency Task Team 2016). A study in China amongst men who have sex with men demonstrated the importance of convenient, safe, discrete, clean HIV testing locations serviced by technically competent, friendly staff (Bien et al. 2015). This raises questions around the scope of quality assurance. We propose that quality assurance should involve not only the technical aspects of performing the test, but also the quality of the surrounding environment, staff and facilities.

### Interventions at Health Facility-Laboratory Interface

#### Intervention 5: Workshops/Meetings/Dialogues Between Health Facilities and Laboratories to Resolve Transport-Related Gaps and Facilitate Return of Results

We propose workshops, meetings or dialogues between health facilities and laboratories to discuss the following:

- Specimen transportation, handling and logging in on arrival at the laboratory and acceptability criteria. Reconciling collected with processed samples will ensure that health facilities are aware which samples were lost in transit. Effective communication between laboratories and health facilities will ensure that rejected samples are promptly re-drawn.
- Blood collection protocols, reasons for delays and challenges in obtaining acceptable laboratory specimens.
- How laboratory HIV test reports collated for a sub-district or district can be distributed to health care workers responsible for initiating ART and referring newly infected patients into care.
- Use of mobile technology to return results to health facilities. A Zambian study conducted at 10 health facilities found that introduction of Short Messaging Services (SMS) systems resulted in a reduction in mean turn-around time (TAT) for result notification to facilities from 44.2 days pre-implementation to 26.7 days post-implementation. The mean TAT for notifying caregivers also dropped from 66.8 days to 35 days (post-implementation) (Seidenberg et al. 2012).
- Furthermore Point-of-Care tests for infant HIV diagnosis (e.g. Alere q™) need to be evaluated in operational settings so that the feasibility and cost implications of scale-up can be investigated.

### At Community Level

#### Intervention 6: Community Leader and Healthcare Worker Advocacy at Creches, Schools, Religious Centres and Through Social Media to Increase Uptake of HIV Testing and Dispel Fatalistic Beliefs About HIV

Home-based HIV Counselling and Testing (HB-HCT) has been highly acceptable with improved HCT coverage and uptake in low and middle-income countries (Matovu et al. 2006; Doherty et al. 2013; Lugada et al. 2010). A randomized controlled trial in Uganda comparing home-based and health facility testing found that 93 % of community members agreed to test in home sites; with 54.6 % of HIV infections identified compared to 27.3 % in villages with health facility-based HIV Counselling and Testing (HCT) (Yoder et al. 2006). HB-HCT involves trained community health workers (CHWs) to offer door-to-door pre-test counseling and HCT services to consenting eligible household members (WHO 2012). Mobile community-based HCT, HB-HCT and school-based HCT are models, which often target hard-to-reach populations such as communities residing in rural and historically disadvantaged settings and individuals who experience difficulties accessing health facilities (Kyaddondo et al. 2012). Such models save time and travelling costs to health facilities (Kyaddondo et al. 2012). Use of rapid testing has simplified HB-HCT implementation (Clark et al. 2008). HB-HCT encourages disclosure and provides family members with an increased opportunity to be informed about each other’s HIV status. Increased knowledge of HIV status among family members has the potential to improve attitudes towards the disease, promote emotional and social support and adherence to antiretroviral therapy (Nuwaha et al. 2012). The feasibility of HB-HCT as a testing approach for adults has been demonstrated, and this approach may be extrapolated to young adults, providing counselling systems are in place (Doherty et al. 2013). A review paper demonstrated that community-oriented strategies such as peer mentors (CHW’s, mentor mothers, traditional chiefs and religious leaders) were highly effective in increasing community based support and improving care and retention along the PMTCT cascade (Marcos et al. 2012). Unlike more specialized healthcare providers; peer mentors were more readily available and could be recruited and trained more efficiently to effectively implement PMTCT interventions. The psychological burden of paediatric HIV infection on parents and children should not be under-estimated (Bernays et al. 2014). Thus action is needed to enhance and sustain peer and community networks and support systems, rehabilitation and counselling and psychological support.

#### Intervention 7: Use of Mobile Communication Technology (m-Health) and Peer/Community Supporters to Maintain Contact with Patients

The Masiluleke project in South Africa also demonstrated success in using mobile technology to encourage HIV/AIDS testing (World Bank 2012). Additionally, a study by Lester et al. (2010) reported significantly increased ART adherence and viral suppression using the SMS texting system to patients.

## Discussion and Conclusions

Given the existing evidence and gaps, we hypothesise that a package of facility, community and family-orientated interventions focusing on improving the demand and supply of paediatric HIV-related services will substantially improve access to paediatric antiretroviral therapy and ongoing care. This could be critical to change the trajectory of the paediatric HIV epidemic and its associated patterns of morbidity and mortality, thus achieving the double dividend of improving HIV-free survival.
